# Lithium-associated hypothyroidism: Reversible after lithium discontinuation?

**DOI:** 10.1192/j.eurpsy.2021.231

**Published:** 2021-08-13

**Authors:** I. Lieber, M. Ott, L. Öhlund, R. Lundqvist, M. Eliasson, M. Sandlund, U. Werneke

**Affiliations:** 1 Sunderby Research Unit, Deparment of clinical science, division of psychiatry, Luleå, Sweden; 2 Medicine, Umeå Univeristy, Department of Public Health and Clinical medicine, Umeå, Sweden; 3 Sunderby Research Unit, Umeå University, Deparment of clinical science, division of psychiatry, Luleå, Sweden; 4 Norrbotten Region, Reserach and Innovation Unit, Luleå, Sweden; 5 Department Of Public Health And Clinical Medicine, Sunderby research Unit, Luleå, Sweden; 6 Division Of Psychiatry, Umeå University, Department of Clinical Sciences, Umeå, Sweden

**Keywords:** lithium, adverse effects, bipolar disorder, hypothyroidism

## Abstract

**Introduction:**

The association between lithium and thyroid dysfunction has long been known. Yet it is not known whether lithium-associated hypothyroidism is reversible, once lithium treatment has been stopped.

**Objectives:**

To determine whether lithium-associated hypothyroidism was reversible in patients who subsequently discontinued lithium.

**Methods:**

Retrospective cohort study in the Swedish region of Norrbotten into the effects and side- effects of lithium treatment and other drugs for relapse prevention (LiSIE). For this particular study, we reviewed medical records between 1997 and 2015 of patients treated with lithium.

**Results:**

Of 1340 patients screened, we identified 90 patients with lithium-associated hypothyroidism who subsequently discontinued lithium. Of these, 27% had overt hypothyroidism at the time when thyroid replacement therapy was initiated. The mean delay from lithium start to thyroid replacement therapy start was 2.3 (SD 4.7) years. Fifty percent received thyroid replacement therapy within 10 months of starting lithium. Of 85 patients available for follow up, 35 (41%) stopped thyroid replacement therapy after lithium discontinuation. Six patients reinstated thyroid replacement therapy subsequently. Only one of these had overt hypothyroidism, occurring 13 days after stopping lithium and 11 days after stopping thyroid replacement therapy.

**Conclusions:**

Lithium-associated hypothyroidism seems reversible in most patients, once lithium has been discontinued. In such cases, thyroid replacement therapy discontinuation could be attempted much more often than currently done. Based on the limited evidence of our study, we can expect hypothyroidism to recur early after discontinuation of thyroid replacement therapy if at all.

**Disclosure:**

MO: scient adv. board member Astra Zeneca Sweden; UW: educ. activities Norrbotten Region: Astra Zeneca, Eli Lilly, Janssen, Novartis, Otsuka/Lundbeck, Servier, Shire and Sunovion. All others: none.

